# Spirometry thresholds for clinical trial eligibility: time for urgent re-evaluation

**DOI:** 10.1136/thorax-2024-222652

**Published:** 2025-09-04

**Authors:** Idan Bokobza, Melanie Le Sayec, Cara Bossley, Rossa Brugha, Jane Carolyn Davies, Charlotte Dawson, Lucy Holt, Dominic A Hughes, Yasmine Needham, Caroline Pao, Gary Ruiz, Shahideh Safavi, Clare Saunders, Nadia Shafi, Nicholas J Simmonds, Michael D Waller, Danie Watson, Gwyneth Davies

**Affiliations:** 1 National Heart and Lung Institute, Imperial College London, London, UK; 2 Clinical Trials Accelerator Platform, London, UK; 3 Royal Brompton & Harefield NHS Foundation Trust, London, UK; 4 King’s College Hospital NHS Foundation Trust, London, UK; 5 Great Ormond Street Hospital for Children NHS Foundation Trust, London, UK; 6 Barts Health NHS Trust, London, UK; 7 King’s College London, London, UK; 8 UCL Great Ormond Street Institute of Child Health, University College London, London, UK

**Keywords:** Cystic Fibrosis, Respiratory Measurement

## Abstract

A common eligibility criterion in respiratory clinical trials is a per cent-predicted forced expiratory volume in 1 second (ppFEV_1_) between 40% and 90%, using the ethnicity-dependent Global Lung Function Initiative (GLI)-2012 spirometry reference equations. International societies now endorse the newer ‘race-neutral’ GLI-Global equations. We quantify the impact on trial eligibility of switching from GLI-2012 to GLI-Global for the UK Cystic Fibrosis Registry (n=8182). In a future trial with a maximum eligibility threshold of ppFEV_1_=90%, the changes in ppFEV_1_ would lead to over 700 people becoming newly ineligible. Urgent review of fixed ppFEV_1_ thresholds is required, with an initial recommendation to widen the range to 30–95% ppFEV_1_ when GLI-Global is implemented to ensure equitable access for people of all ethnicities. There is also a wider need to review the use of fixed ppFEV_1_ spirometry limits for trial eligibility.

## Introduction

 Respiratory clinical trials regularly use eligibility criteria containing fixed cut-off values for spirometry indices such as per cent-predicted forced expiratory volume in 1 second (ppFEV_1_). Trials involving people with cystic fibrosis (pwCF) generally require participants to have a ppFEV_1_ between 40% and 90%. The published median ppFEV_1_ for the UK CF population is 85%,^1^ using Global Lung Function Initiative (GLI)-2012 equations,^2^ meaning many pwCF are near the upper ppFEV_1_=90% threshold, with this a common barrier to trial participation.^3^

Most clinical trials with spirometry eligibility thresholds use GLI-2012 to calculate ppFEV_1_. GLI-2012 includes ethnicity as an input variable required to generate ppFEV_1_. However, the American Thoracic Society and European Respiratory Society now endorse the newer GLI-Global^4^ ‘race-neutral’ equations to reduce the false view of fixed differences between ethnicities.^5^ Any change in ppFEV_1_ following application of GLI-Global to an individual’s spirometry values is a consequence of the equation rather than any pathological or physiological process. There is no change in that individual’s risk or potential to demonstrate intervention efficacy.

## Objective

Using CF as an example, we investigated the impact of changing from GLI-2012 to GLI-Global equations on clinical trial eligibility.

## Methods

We analysed data from pwCF entered on the UK Cystic Fibrosis Registry (UK CF Registry) with a 2022 annual review spirometry recorded, including those with a ppFEV_1_<150%, and with data available for all variables required for calculating GLI-2012 ppFEV_1_ (n=8182). Data request approvals were granted by the UK CF Registry Research Committee.

The variables available were: FEV_1_ (litres), sex, height (centimetres), age and ethnicity (White, Black, Northeast Asian, Southeast Asian, Other/Mixed). These were inputted into the GLI-calculator (https://gli-calculator.ersnet.org/) to provide ppFEV_1_ values for GLI-2012 and GLI-Global. For the GLI-Global calculator, the ethnicity category is set to ‘race-neutral’.

ppFEV_1_ values for each of the equations were analysed according to hypothetical trial inclusion criteria of (a) minimum ppFEV_1_=40% and (b) maximum ppFEV_1_=90%. We quantified changes in trial eligibility status when moving from GLI-2012 to GLI-Global.

## Results

Our cohort had a mean (SD) GLI-2012 ppFEV_1_ of 80.6% (23.4%) and median (IQR) age of 24.8 (14.2–35.7) years. Table 1 shows the number of individuals within each ethnicity on the 2022 UK CF Registry as required for GLI-2012 input, and the corresponding mean change in ppFEV_1_ when switching to GLI-Global. The largest change in ppFEV_1_ was for black persons with a mean *decrease* of 7.6%. White persons were the largest group and demonstrated a mean ppFEV_1_ *increase* of 4.4%.

**Table 1:** Change in ppFEV_1_ on switching from GLI-2012 to GLI-Global equations according to ethnicity.

Ethnicity	People (n)	Mean change in ppFEV_1_ between GLI-2012 and GLI-Global (%)	Range of change in ppFEV_1_ (%)
White	7790	+4.4	−4 to +12
Black	21	−7.6	−18 to −1
Southeast Asian	2	+4.0	+2 to +6
Northeast Asian	0	–	–
Other/mixed	369	−1.3	−9 to +3
Combined total	8182	+4.1	−18 to +12

A positive number indicates an increase in ppFEV_1_ when moving to GLI-Global. Ethnicity as recorded on the UK CF Registry was grouped according to the ethnicity categories for the GLI-2012 equations: White, Black, Southeast Asian, Northeast Asian, Other/Mixed.

GLI, Global Lung Function Initiative; ppFEV_1_, per cent-predicted forced expiratory volume in 1 second.

**Figure 1:**
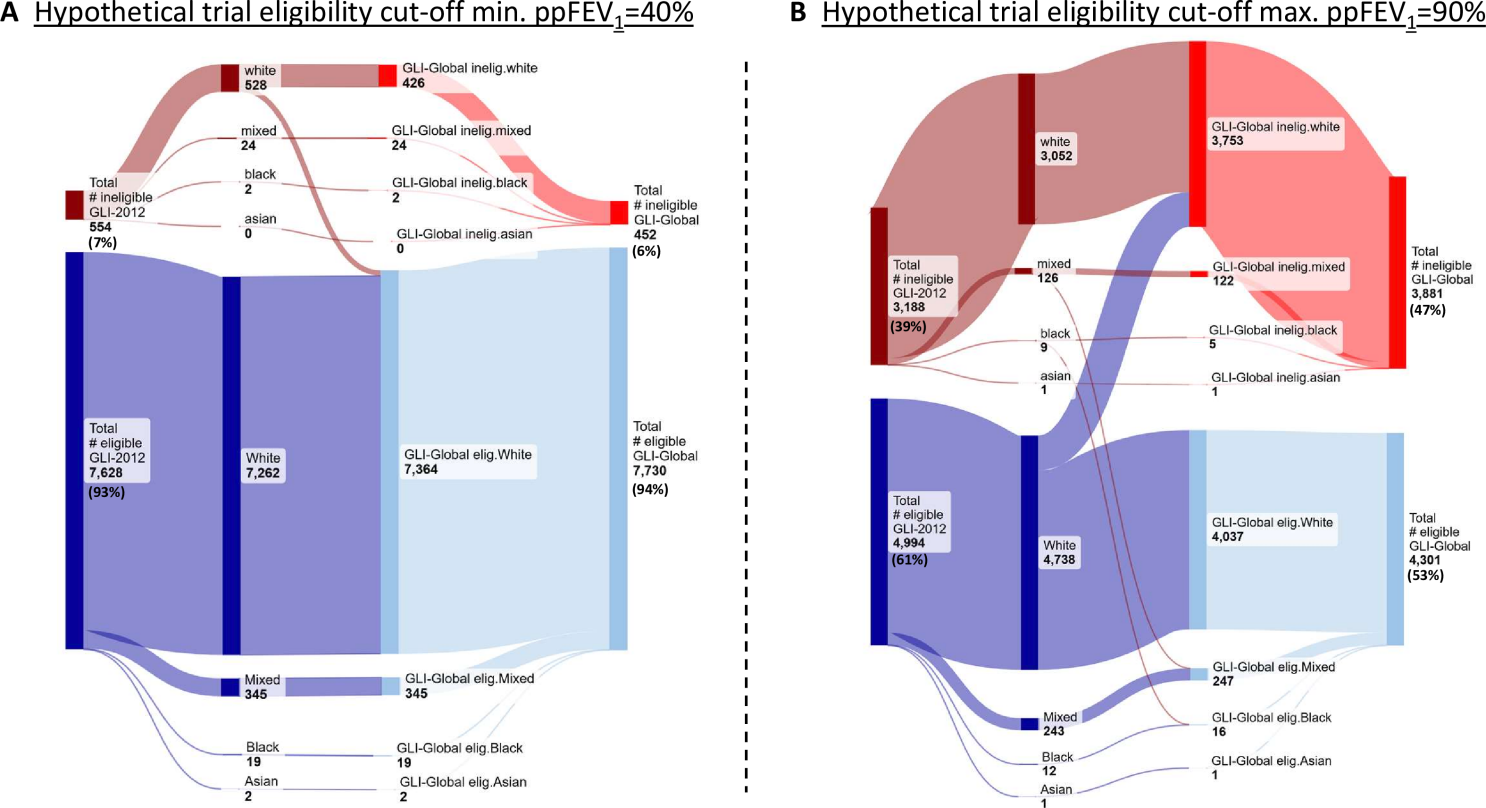
Sankey diagrams illustrating changes in eligibility when moving from Global Lung Function Initiative (GLI)-2012 to GLI-Global for a trial with (A) a minimum cut-off ppFEV_1_=40% and (B) a maximum cut-off ppFEV_1_=90%. Dark blue and light blue represent those eligible using GLI-2012 and GLI-Global, respectively. Dark red and light red/orange represent those ineligible using GLI-2012 and GLI-Global, respectively. Asian denotes Southeast Asian. elig, eligible; GLI, Global Lung Function Initiative; inelig, ineligible; ppFEV_1_, per cent-predicted forced expiratory volume in 1 second.

Switching equations influenced clinical trial eligibility for protocols using ppFEV_1_ eligibility cut-offs of 40% and 90%. Figure 1A illustrates how 102 persons, all of white ethnicity, would become newly eligible when moving to GLI-Global for a trial with a cut-off of minimum ppFEV_1_=40%.

The greatest impact was for trials using a maximum ppFEV_1_=90% cut-off (figure 1B). Moving to GLI-Global equations would result in a net decrease of 693 eligible for clinical trials to 4301 (701 white persons newly ineligible, with four mixed and four black persons newly eligible).

**Figure 2:**
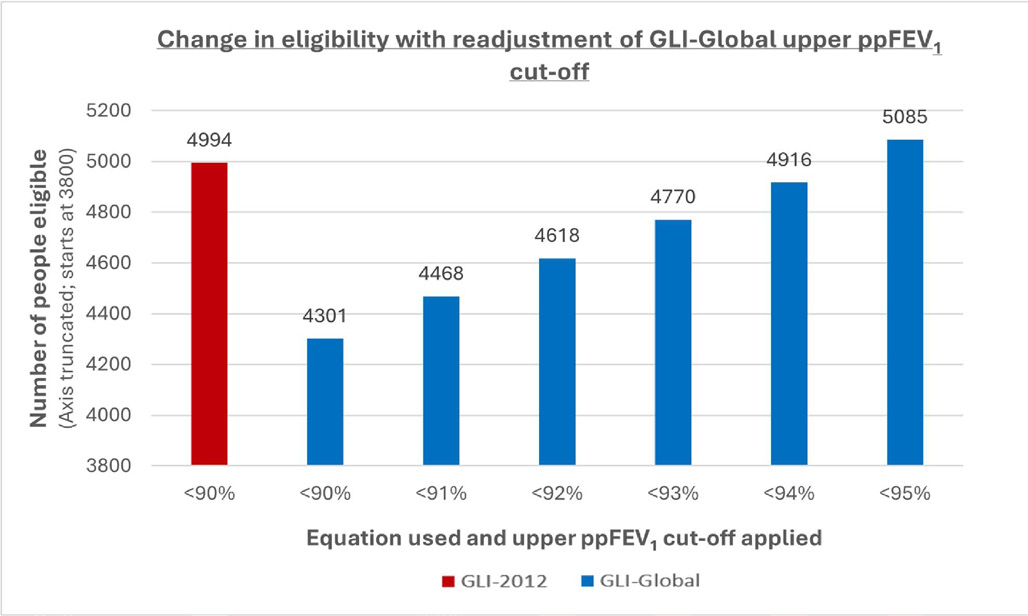
Bar chart showing the number of people within the UK CF Registry potentially eligible for a clinical trial using either GLI-2012 (red) or GLI-Global (blue), according to different upper ppFEV_1_ cut-offs. The Y-axis starts at 3800 to aid visualisation of the changes in eligibility. GLI, Global Lung Function Initiative; ppFEV_1_, per cent-predicted forced expiratory volume in 1 second.

Figure 2 shows the increasing trend in eligibility with the application of a new upper GLI-Global ppFEV_1_ cut-off. A 95% threshold would increase eligibility from 4301 to 5085.

## Discussion

For pwCF in the UK, trial eligibility numbers were significantly impacted by moving to GLI-Global, despite this being independent of a change in the population’s health. This illustrates how fixed ppFEV_1_ cut-offs engrained in trial protocols need to be re-evaluated when more appropriate equations are published. Diao *et al*^6^ recently described other downstream impacts with a move to GLI-Global, including on disease classifications, but did not explore trial eligibility.

Due to our population demographics, the largest change in eligibility is seen in the white CF population, with the formula switch newly excluding >700 people from participation due to the upper ppFEV_1_=90% threshold. However, it is crucial to note that the largest mean change in ppFEV_1_ was in people of black ethnicity with a decrease in ppFEV_1_ of 7.6%, although in a small sample size. This decrease is in keeping with Rosenfeld *et al*^7^ which includes a larger cohort of people of black ethnicity. Switching to GLI-Global would therefore substantially disadvantage people of black ethnicity near the minimum ppFEV_1_=40% threshold.

These findings suggest the need for updated wider thresholds as a first immediate step to allow continued access to clinical trials for studies using ppFEV_1_ as an eligibility criterion. With the largest mean increase in ppFEV_1_ at 4.4% and an increase negatively affecting people near the existing ppFEV_1_=90% upper threshold, we suggest increasing this to 95%. With the largest decrease being in people of black ethnicity at 7.6%, a decrease in the threshold to ppFEV_1_=30% is also recommended. We appreciate that any ppFEV_1_ thresholds introduce age and sex bias, and that z-scores are more appropriate statistically for summarising a person’s FEV_1_ status. Our focus has been on ppFEV_1_ to reflect clinical trial protocols.

Increasing the upper threshold to ppFEV_1_=95% risks ceiling effects, with trials possibly failing to detect lung function improvements in people with near-normal ppFEV_1_. However, the threshold increase is merely to include a similar population already included with GLI-2012. Nevertheless, ceiling effects may be particularly relevant in our CF population where the introduction of cystic fibrosis transmembrane conductance regulator (CFTR) modulators has already significantly improved the lung function in those eligible and with access to these therapies. Statistical considerations such as subgroup analyses of those in this higher bracket can be used as a countermeasure. There is also evidence that efficacy can still be demonstrated with a baseline ppFEV_1_>90% with GLI-Global^8^ or GLI-2012.^9^

Alternative outcome measures are also increasingly used, such as Lung Clearance Index (LCI) measured by multiple breath washout technique. LCI is considered a more sensitive measure of lung function in early disease^10^ and may therefore still show treatment effects in those with milder disease.

At the other end of the spectrum, patient safety concerns may be raised if suggesting the lower ppFEV_1_ threshold is 30% rather than 40%. However, again, the aim is to be inclusive. Furthermore, Elborn *et al*^11^ also illustrate that a ppFEV_1_ between 30% and 40% could be considered safe in a CFTR modulator trial, although in a small subgroup analysis including 81 participants. Finally, other criteria for stability should be prioritised, such as investigator discretion taking into account the patient’s full clinical context.

This lower threshold is critical to ensure equity of access across ethnic minorities, especially in the CF population, with these populations more likely to have *CFTR* variants ineligible for CFTR modulators, and at even greater need to access new medications on clinical trials. In CF, this includes the *CFTR* mRNA and DNA studies currently enrolling participants.

The implications of this study go beyond CF and are relevant to any trials using ppFEV_1_ as eligibility criteria, including in chronic obstructive pulmonary disease (COPD) and asthma. We appreciate that not all trials involving spirometry use ppFEV_1_ values, and those that do may not include the same thresholds.

## Conclusion

GLI-Global is rightly endorsed by international societies. With implementation, an opportunity exists to review fixed ppFEV_1_ thresholds, relevant beyond CF in promoting equitable access to clinical trials. We clearly demonstrate the impact if thresholds are not urgently revised, with hundreds of people potentially excluded from participation. New thresholds of ppFEV_1_ 30–95% would provide an immediate pragmatic solution ensuring continued access to clinical trials to people of all ethnicities despite this formula change. This should urgently be followed by research into more appropriately quantifying individual-level risk and benefit following therapeutic intervention, and whether fixed cut-offs are appropriate at all. We ask the respiratory trials community, from the patient community to sponsors, to heed this call and respond.

## Supplementary material

10.1136/thorax-2024-222652 online supplemental file 1
