# Supplementary material for: Spirometry thresholds for clinical trial eligibility: time for urgent re-evaluation
Source: Thorax. 2025 Sep 4;80(11):e222652. doi: 10.1136/thorax-2024-222652 (PMC12573418; doi:10.1136/thorax-2024-222652)
Supplement: online supplemental file 1 [file thorax-80-11-s001.pdf]

Spirometry thresholds for clinical trial eligibility: time for urgent re-evaluation

Bokobza I, et al. Thorax 2025 DOI: 10.1136/thorax-2025-222652

Background

• GLI-Global is the newly endorsed spirometry reference equation.

• We investigated $ppFEV_1$ changes for the UK CF population when changing from GLI-2012 to GLI-Global and its impact on clinical trial access using common eligibility thresholds.

• Each ethnicity is affected differently:

Ethnicity	Number of people	Mean change in $ppFEV_1$ between GLI-2012 & GLI-Global (%)
White	7790	+ 4.4
Black	21	- 7.6
S.E Asian	2	+ 4.0
Mixed	369	- 1.3
Total	8182	+ 4.1

Table 1. A '+' change indicates an increase in $ppFEV_1$ when moving to GLI-Global. Population assessed is from the UK Cystic Fibrosis registry.

Results

A. Hypothetical trial eligibility cut-off min. $ppFEV_1$=40%

B. Hypothetical trial eligibility cut-off max. $ppFEV_1$=90%

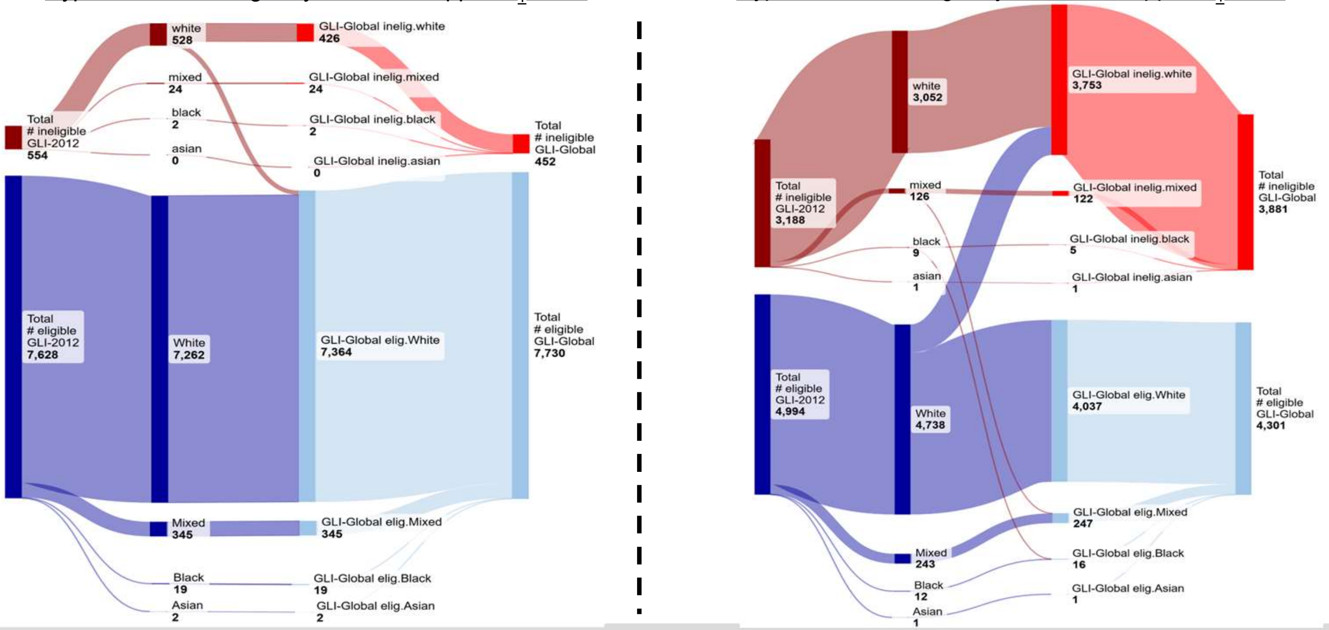

Conclusion

To maintain access to clinical trial participation, we suggest new $ppFEV_1$ thresholds of minimum 30% and maximum 95% in the first instance. There is also a wider need to review the use of fixed $ppFEV_1$ spirometry limits for trial eligibility.

Thorax thorax.bmj.com @ThoraxBMJ British Thoracic Society BMJ Group
